# Clean birth and postnatal care practices to reduce neonatal deaths from sepsis and tetanus: a systematic review and Delphi estimation of mortality effect

**DOI:** 10.1186/1471-2458-11-S3-S11

**Published:** 2011-04-13

**Authors:** Hannah Blencowe, Simon Cousens, Luke C Mullany, Anne CC Lee, Kate Kerber, Steve Wall, Gary L Darmstadt, Joy E Lawn

**Affiliations:** 1 London School of Hygiene and Tropical Medicine, London, WC1E 7HT, UK; 2 Department of International Health, Johns Hopkins Bloomberg School of Public Health, Baltimore, MD 21205, USA; 3 Saving Newborn Lives/Save the Children, Cape Town, South Africa; 4 Current address: Family Health Division, Global Health Program, Bill and Melinda Gates Foundation, Seattle, WA 98102, USA; 5 Health Systems Strengthening Unit, Medical Research Council, Cape Town, South Africa

## Abstract

**Background:**

Annually over 520,000 newborns die from neonatal sepsis, and 60,000 more from tetanus. Estimates of the effect of clean birth and postnatal care practices are required for evidence-based program planning.

**Objective:**

To review the evidence for clean birth and postnatal care practices and estimate the effect on neonatal mortality from sepsis and tetanus for the Lives Saved Tool (LiST).

**Methods:**

We conducted a systematic review of multiple databases. Data were abstracted into standard tables and assessed by GRADE criteria. Where appropriate, meta-analyses were undertaken. For interventions with low quality evidence but a strong GRADE recommendation, a Delphi process was conducted.

**Results:**

Low quality evidence supports a reduction in all-cause neonatal mortality (19% (95% c.i. 1–34%)), cord infection (30% (95% c.i. 20–39%)) and neonatal tetanus (49% (95% c.i. 35–62%)) with birth attendant handwashing. Very low quality evidence supports a reduction in neonatal tetanus mortality with a clean birth surface (93% (95% c.i. 77-100%)) and no relationship between a clean perineum and tetanus. Low quality evidence supports a reduction of neonatal tetanus with facility birth (68% (95% c.i. 47-88%). No relationship was found between birth place and cord infections or sepsis mortality. For postnatal clean practices, all-cause mortality is reduced with chlorhexidine cord applications in the first 24 hours of life (34% (95% c.i. 5–54%, moderate quality evidence) and antimicrobial cord applications (63% (95% c.i. 41–86%, low quality evidence). One study of postnatal maternal handwashing reported reductions in all-cause mortality (44% (95% c.i. 18–62%)) and cord infection ((24% (95% c.i. 5-40%)).

Given the low quality of evidence, a Delphi expert opinion process was undertaken. Thirty experts reached consensus regarding reduction of neonatal sepsis deaths by clean birth practices at home (15% (IQR 10–20)) or in a facility (27% IQR 24–36)), and by clean postnatal care practices (40% (IQR 25–50)). The panel estimated that neonatal tetanus mortality was reduced by clean birth practices at home (30% (IQR(20–30)), or in a facility (38% (IQR 34–40)), and by clean postnatal care practices (40% (IQR 30–50)).

**Conclusion:**

According to expert opinion, clean birth and particularly postnatal care practices are effective in reducing neonatal mortality from sepsis and tetanus. Further research is required regarding optimal implementation strategies.

## Background

More than half a million newborns are estimated to die each year from serious neonatal infections, accounting for about 15% of all neonatal deaths globally [1]. The most vulnerable time for both the mother and newborn is during birth and in the hours and days immediately after childbirth. Around 75 percent of neonatal deaths occur during the first week of life, with the majority in the first 48 hours [2], which is also the period of highest risk for mothers [3]. In populations with very high neonatal mortality, up to half of neonatal deaths may have an infectious cause [4,5].

It is estimated that 30-40% of infections resulting in neonatal sepsis deaths are transmitted at the time of childbirth and have early onset of symptoms (developing during the first 72 hours after birth) [6,7]. In low income countries, about 60% of births occur without a skilled attendant, most of these at home [8]. Worldwide, 60 million births happen outside facilities and even for facility births hygienic practices may be sub-optimal.

In addition many neonatal deaths due to tetanus and other infections are acquired postnatally [9,10]. The unhealed umbilical cord is an important portal for local and invasive infections during this period and is rapidly colonised by bacteria from the maternal genital tract and then from the environment. Localised umbilical infection (omphalitis) can spread to the abdominal wall, the peritoneum, or through the umbilical or portal vessels leading to systemic sepsis, which, if untreated, has a high case-fatality rate [11]. Omphalitis with redness extending to the abdominal wall was associated with a 46% increased risk of mortality in rural Nepal [12].

The global burden of neonatal tetanus has reduced over from over 600,000 neonatal deaths in 1990 to fewer than 60,000 in 2008 [1,13]. Increased tetanus toxoid vaccination coverage and hygienic intrapartum and postnatal practices, particularly cord care, are important contributing factors [14,15]. In addition to variation in immunization coverage, intrapartum and postnatal practices may explain much of the local variation in incidence of tetanus [16-18]. Clean birth practices have been associated with dramatic reductions in the incidence of neonatal tetanus in the absence of immunization, for example in industrialized countries where tetanus was virtually eliminated before the vaccine was introduced and. in China, training of traditional birth attendants (TBAs) and providing them with a ‘clean birth kit’ in the 1950s led to a reduction in neonatal tetanus rates from 32/1000 in 1948 to 2/1000 in 1961 [19].

**Figure 1:**
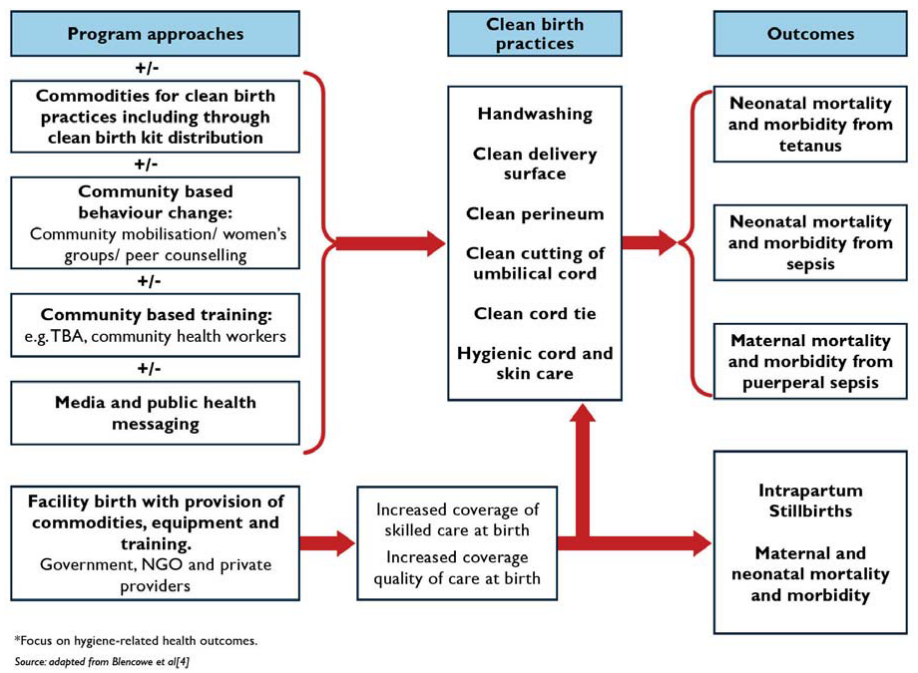
Conceptual framework of potential role of implementation mechanisms, including kits on clean practices at birth and effects on neonatal and maternal outcomes

Hygienic behaviours during childbirth and during the early postnatal period are variably defined. In this paper, we define clean birth and postnatal care practices in accordance with World Health Organisation’s (WHO) “six cleans” - hand washing of birth attendant before birth, clean birth surface, clean perineum, cutting of the umbilical cord using a clean implement, clean cord tie, and a clean cloth for drying (Figure 1). These practices may be influenced by a number of programmatic approaches including behaviour change communications, commodity provision, or training of attendants, or combinations of these, and the context may involve a facility birth or a home birth. Hand-washing with soap results in a large reduction in hand contamination, even when washed with unclean water [20], and birth attendant and maternal hand washing have been associated with reductions in neonatal mortality [21]. However cultural factors frequently govern practices and may influence willingness to adopt new clean practices [22-24]. Many populations commonly rub potentially harmful substances on the umbilical cord or skin despite WHO recommendations for dry cord care [11,25-29]. Chlorhexidine, a broad-spectrum topical antiseptic, has residual effect for up to 72 hours and may be a useful adjunct to basic clean practices in home and facility settings, especially where unhygienic applications to the cord are common.

Although such hygienic practices at birth and during the postnatal period are accepted as a standard of care, there is very limited evidence regarding their effect to guide policymakers in the potential mortality effect size. Individually randomized trials of hygienic versus unhygienic practices would clearly be unethical, and thus the quality of evidence will always be limited. There is increasing evidence from cluster randomized trials in South Asia of the significant effect on neonatal mortality of postnatal care practices, including clean practices, [26,30-35]. However all these studies involve packages with varying intervention content, often with antenatal and intrapartum care, and in several cases also providing curative care for infections at home in the postnatal period. The relative importance and contribution of each component is difficult to determine. An additional challenge is that evaluations often consider intermediate outcomes such as sepsis morbidity or omphalitis, which are variably defined.

This review does not include other interventions to reduce deaths from neonatal infections, such as tetanus toxoid immunization [15], antibiotics for prolonged rupture of membranes, early and/or exclusive breastfeeding or skin-to-skin care, or case management of neonatal sepsis since these topics are reviewed elsewhere [36-39]. Delivering in a facility with a skilled attendant and access to emergency obstetric care has the potential to reduce stillbirths [40], maternal deaths and also neonatal deaths from causes other than infection [41], and these outcomes are also considered in other reviews.

### Objective

The objective of this review is to estimate, for use in the Lives Saved Tool (LiST), the effect of clean practices at birth and during the postnatal period on all-cause neonatal mortality, cause-specific mortality from sepsis and tetanus, and infection-related morbidity (e.g. sepsis and omphalitis).

## Methods

This review uses an adaptation of the GRADE approach and is designed to provide estimates for use in LiST which models cause-specific deaths averted by increases in coverage of effective interventions. Details of the general review methods, the adapted GRADE approach and the LiST model have been described elsewhere [42].

### Searches

**Figure 2:**
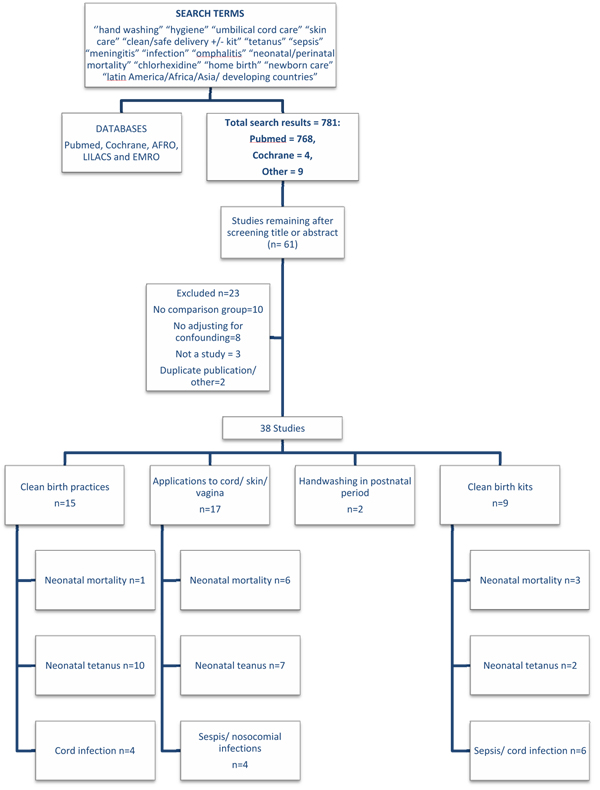
Search strategy and results

We systematically reviewed the published literature to identify studies of clean birth and postnatal practices for the prevention of neonatal sepsis and tetanus mortality and morbidity from 1980 until February 2010. We searched PubMed, EMBASE, Cochrane Libraries, and all WHO Regional Databases and included publications in any language [42]. Combinations of the following search terms were used: *“clean/safe birth/delivery* +*/- kit”*, *“tetanus*,*”*, *“sepsis*,*” “meningitis*,*”*, *“infection*,*” “omphalitis/oomphalitis”*, *“hygiene*,*” “hand washing*,*” “umbilical cord care*,*” “skin care*,*” “neonatal/perinatal mortality”*, *“newborn care”*, *”chlorhexidine”*, *“home birth”*, *“Latin America/Africa/Asia or developing country” limited to newborn 0 – 28 days*.(Figure 2, and web annex for details of strategy) After initial screening of titles and abstracts we reviewed full text publications of potentially eligible studies. Snowball searching was used whereby literature referenced in key papers was also searched, including grey literature.

Studies were considered that reported on neonatal/perinatal mortality, neonatal tetanus, sepsis, and meningitis or cord infection.

### Inclusion/exclusion criteria

We applied the PICO format (Population, Intervention, Comparison, and Outcome) as follows. The *population* of interest was neonates. The *interventions* being reviewed are defined below and in table 1 and were *compared* to the absence of clean birth and postnatal practices. The *outcomes* of interest were overall neonatal mortality, neonatal mortality from sepsis or tetanus, and neonatal morbidity (sepsis or cord infection) as defined below. We considered both randomised trials and observational studies (Figure 2). We excluded studies not fulfilling the inclusion criteria and any duplicate reports of trials or studies, studies that included clean practices as part of a package of multiple co-interventions, studies based in specialised populations and case-control, cohort and cross-sectional studies which made no attempt to adjust for confounding. Possible adverse effects of the interventions were not addressed as part of this review.

**Table 1:** Definitions of interventions considered regarding clean practices at birth and in the postnatal period

1) **Clean birth practices:**
a. Place of birth (facility or home) b. Hand washing (birth attendant before birth, with soap) c. Clean perineum (washed prior to birth) d. Clean birth surface (new/ clean plastic sheet or mat) e. Cutting of the umbilical cord using a clean implement (new or boiled blade, or clean scissors) f. Clean cord tying (using a new, clean thread to tie cord or cord clamp)
2) **Hygienic cord and skin care (mainly postnatal):**
a. Combined chlorhexidine cleansing of the birth canal prior to birth and/or full body newborn cleansing immediately after birth b. Chlorhexidine to the cord c. Other antimicrobial applications to the cord d. Avoidance of harmful cord applications e. Skin applications and emollients
3) **Other clean postnatal newborn care practices:**
a. Hand washing (maternal during the postnatal period, with soap)
b. Exclusive breast feeding (considered in a separate review)

### Intervention definitions

Interventions considered in this review include clean birth practices at home or in a health facility, including hand washing, clean cord cutting and tying, clean birth surface, clean perineum, topical antiseptic applications to the cord and skin, and clean birth kit use. In addition clean postnatal newborn care practices and applications to the birth canal, umbilical cord or skin were reviewed (Table 1). The effect of a single-use package of commodities designed to facilitate a clean birth at home or in a health facility (a clean birth kit (CBK)) was considered. A CBK was defined as a disposable package containing at least the minimum commodities required to facilitate clean cutting and tying of the umbilical cord e.g. a clean blade, clean cord tie or clamp.

From a programmatic viewpoint, how to change complex behaviours and cultural norms that govern practices around the time of birth is of great importance, but is not the focus of this paper (Figure 1).

### Neonatal outcomes definitions

We used cause of death definitions consistent with the Child Health Epidemiology Reference Group (CHERG) based on ICD 10 rules [1,43]. Our outcomes of interest were neonatal tetanus or neonatal sepsis (sepsis/septicaemia, meningitis). When no cause-specific data were available, all-cause neonatal mortality was considered. When no effect on neonatal mortality was available, the following neonatal morbidity outcomes were considered: a) neonatal sepsis; b) omphalitis / cord infection. For both of these morbidities, standardisation of diagnosis both within and between studies is problematic. Studies using different clinical definitions were included in the review, and differences between case definitions are described where relevant (Table 2).

**Table 2:** Cord infections and sepsis definitions used in the included studies

Neonatal Outcome and Study	Definition Used
**Neonatal Sepsis Mortality**	

Bakr 2005	Positive microbiological cultures or clinical and laboratory criteria very suggestive of sepsis (e.g., temperature instability, poor feeding, apnea, irregular respiration, positive C-reactive protein [CRP] and micro-erthrocyte sedimentation rate [micro-ESR]) and died in first 28 days of life.
Taha 1997	Paediatricians diagnosed on the basis of clinical criteria of temperature > 38.0°C, poor feeding, and apnoea or irregular respiration and died in first 28 days of life.
Cutland 2009	Culture-confirmed or clinical sepsis on the basis of clinical and laboratory signs and died in first 28 days of life.
Mullany 2006	Presence of 2 or more of the following signs or symptoms: (1) caregiver's report of fever; (2) vomiting more than half of feeds; (3) unconsciousness; (4)bulging fontanelle; (5) feeding difficulty (not able to suck before death or feeding less thannormal); (6) skin or umbilical cord infection (pus discharge from the cord stump); (7) jaundice;and (8) difficulty breathing and either rapid breathing or chest indrawing and died in first 28 days of life.

**Neonatal Sepsis Incidence**	

Cutland 2009	Culture-confirmed or clinical sepsis on the basis of clinical and laboratory signs
Saleem 2007	Neonates who were severely ill according to Integrated Management of Childhood Illness AND had a clinical presentation, maternal history, and involvement of at least one organ system and laboratory findings; or a maternal history supporting infection; or had no evidence of a nonseptic condition to account for their condition
Garner 1994	Based on clinical assessment of study physician

**Cord Infection / Omphalitis**	

Tsu 2000	Used colour photos of normal and infected cord stumps and questions re redness and pus; interviewer assessment and final decision by neonatologist review of this info (rating it as “definite”,“probable”, “possible”, or “unlikely”)
Mullany 2006/7	“Mild” redness (or swelling) was limited to the cord stump, while “moderate” or “severe” was defined as inflammation extending to the skin at the base of the stump (i.e., <2 cm extension onto the abdominal skin) or affecting an area 2 cm or more from the cord, respectively
Winani 2007	Inspection of umbilical stump by village health worker for signs of possible infection, including erythema, tenderness of tissues surrounding the cord, pus discharge, or smelly or moist stump. Diagnosis confirmed by physician.
Darmstadt 2009	Redness, oozing, or bleeding of umbilical stump

### Abstraction, analyses and summary measures

All studies meeting the inclusion criteria were abstracted onto a standardised abstraction form for each outcome of interest [42]. Each study was assessed and graded according to the CHERG adaptation of the GRADE technique [44]. The evidence was summarised by outcome including a qualitative assessment of study quality and sources of bias adapted from the Cochrane review handbook [42]. CHERG Rules for Evidence Review were applied to the collective evidence to provide an estimate for reduction in neonatal mortality from sepsis and neonatal tetanus [42]. Meta-analyses were conducted when appropriate with STATA version 10.0 statistical software [45]. Heterogeneity was assessed using I^2^ and the chi-squared test. When evidence of heterogeneity was present (p<0.10), a random effects model was used, otherwise a fixed effect was assumed. Summary risk ratios and corresponding 95% confidence intervals (CI) are reported.

### Delphi expert consensus panel

For interventions with low or very low quality evidence, we sought expert consensus via the Delphi method [46]. The questionnaire was developed by JL, HB, ACL and Wendy Graham and piloted prior to use. The Delphi form included the background and aims of the Delphi process and requested eight different neonatal effect estimates and 2 maternal effects estimates (Additional File 1). Respondents were allowed the option of anonymous response. The median response, range, and inter-quartile range were determined for each question. Consensus was defined a priori as having been achieved when the inter-quartile range of responses to a given question was ≤ 30%. The process was repeated until consensus was reached.

## Results

Our searches identified 778 records, and snowball searching identified a further three papers. (Figure 2) After initial screening of the title or abstract, we reviewed 61 papers for data on the outcome measures of interest. Twenty three papers did not fulfil the inclusion criteria and were excluded (Figure 2). Thirty eight papers were included in the final database (Additional File 2). Four relevant Cochrane Reviews were identified. One review of topical umbilical cord care at birth found no evidence of benefit of topical antibiotic or antiseptic applications in high income countries, but no data from low income settings were included in the review [47]. The second, a study of topical ointment for preventing infection in preterm infants, reviewed four facility-based studies from high income countries and concluded that prophylactic topical ointments should not be used for premature babies in high-income settings due to an increased risk of nosocomial and other infections in the treated group [48]. The final two reviews examined the effect of vaginal cleansing with chlorhexidine on Group B streptococcus [49] and other neonatal infections in high-income countries [50]. Whilst cleansing reduced colonisation with Group B streptococcus, no other benefits in terms of sepsis morbidity or mortality were observed.

The remainder of this review focuses on studies carried out in low/middle income countries where the effect of the clean practices is likely to be larger, and the evidence more applicable to decision makers in the countries using the LiST tool. The order of the results section follows the list in table 1

### 1. Evidence for the effect of clean birth practices

#### a. Place of birth

**Figure 3:**
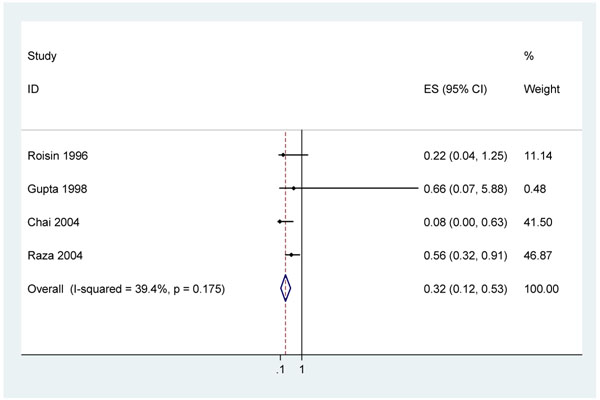
Meta-analysis of neonatal tetanus variation with facility birth compared to non-facility birth controlling for major confounders (maternal education, tetanus toxoid immunization, care knowledge/ practices)

Most studies of clean birth practices are carried out in populations with high rates of home birth. Direct evidence for the effect of clean birth practices in a facility compared to clean birth practices at home on overall neonatal mortality or sepsis-related mortality is complex to assess given multiple confounders. Four studies reported a reduced risk of neonatal tetanus associated with facility delivery after adjusting for potential confounders [adjusted odds ratio (aOR) 0.56 (95% confidence interval (CI) 0.32 – 0.91)[51], aOR 0.22 (95% CI 0.04 – 1.25) [52], aOR 0.08 (95% CI 0.003 – 0.63) [53], aOR 0.66 (95% CI 0.07 – 5.88) [54]]. (Figure 3)

Another study reported a crude neonatal mortality OR of 0.22 (95% CI 0.08 – 0.71) [55], but reported no association after adjusting for potential confounders. Multivariate analysis of a study from Tanzania indicated a reduction in cord infection if a broad definition (pus with any degree of redness) was used [adjusted rate ratio (aRR) 0.38 (95% CI0.19 – 0.78)] amongst hospital births when compared to home births [56]. However, when a more restrictive definition was used (moderate or severe redness), an increase in cord infection was seen in hospital births [aRR 2.05 (95% CI 1.12 – 3.72)]. Three further studies found no difference in cord infection rates between home and facility deliveries [57-59].

#### b. Hand washing of birth attendant with soap before birth

**Figure 4:**
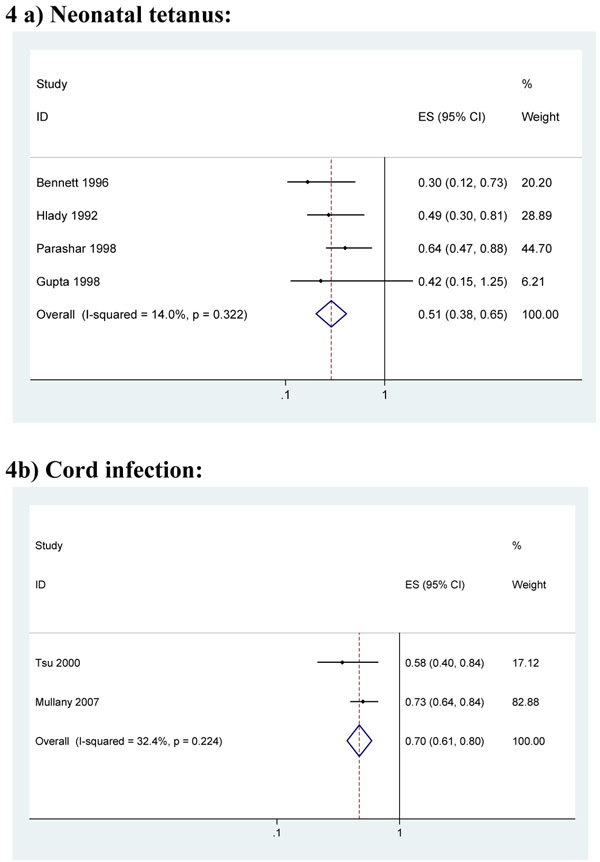
Meta-analysis of the effect of birth attendant hand washing before birth: **4a)** On neonatal tetanus **4b)** On cord infection

Eight observational studies that adjusted for confounding were identified. Four community-based case-control studies and one cohort study reported the effect of birth attendant hand-washing on tetanus-specific neonatal mortality in rural populations. The aORs were all below 1, consistent with a protective effect of hand-washing (Figure 4a) [60-63]. Combining the four studies which provided point estimates and CIs resulted in a pooled effect estimate of 0.51 (95% CI. 0.38 – 0.65). (Figure 4a) The final study provided a point estimate of aOR=0.19 (p<0.001) [64]

One large cohort study (n=23,662 with 713 deaths) reported lower neonatal mortality associated with reported birth attendant hand-washing with soap and water (aRR=0.81: 95% CI 0.66 – 0.99) [21]. No cases of neonatal tetanus were documented in this study population with near universal coverage of antenatal tetanus toxoid coverage and access to CBKs. This study and a further cohort study from Nepal also reported reductions in the incidence of cord infection associated with birth attendant hand-washing: aRR=0.73 (95% CI 0.64 – 0.84) [11] and aRR=0.58 (95% CI 0.40 – 0.84) [58], respectively. (Figure 4b)

#### c. Clean perineum (washed prior to birth)

Two case-control studies reported no association after adjustment between cleaning the perineum, with soap and water, and reduction in the incidence of neonatal tetanus [51,65]. No evidence on the effect of cleaning of the perineum on sepsis or infection was identified.

#### d. Clean birth surface (new or clean sheet or mat)

Two case-control studies controlling for potential confounders examined the association between using a clean plastic sheet as a birth surface and incidence of neonatal tetanus and reported aOR=0.31 (95% CI 0.10 – 0.91)[66] and aOR=0.03 (95% CI 0.002 – 0.34) [55]. One cohort study from Tanzania, with nearly universal kit use in the study population, did not find evidence of a protective effect of using a clean plastic sheet on incidence of cord infection after adjusting for potential confounding factors. However, the adjusted relative risk is not presented [56].

#### e. Cutting of the umbilical cord using a clean implement

Seven observational studies (4 case-control, 2 cohort, 1 adopter vs. non-adopter) were identified which examined the association of using a clean implement (a new/ boiled/ sterile blade or scissors) to cut the cord with neonatal sepsis or tetanus.

Five of these studies from Asia and one from Senegal examined the effect on neonatal tetanus incidence or mortality. Two case-control studies and one cohort study which sought to adjust for confounders reported strong evidence of lower neonatal tetanus mortality associated with use of clean cord cutting tools [aOR=0.3 (95% CI 0.13 – 0.62)[60], aOR=0.4 (95% CI 0.24 – 0.66) [61], and aOR0.25 (95% CI 0.08 – 0.75)][63].Three studies did not find strong statistical evidence of an association between tetanus and the use of an old razor blade or scissors versus a new blade after controlling for potential confounders but no parameter estimates were reported [51,64,66].

One study of clean birth kits from Tanzania examined the effect of the use of a new blade on cord infection. There was no reduction in cord infection associated with the use of new blades [aOR=1.1 (0.43 – 3.05)]. However, the level of new blade use was >95%, and those not using new blades may have used boiled blades [59].

#### f. Clean cord tying

Four case-control studies examined the association between use of a ‘new clean thread’ to tie the umbilical cord at birth and neonatal tetanus incidence or mortality. A study from Uganda using hospital-based cases reported an aOR of 0.1 (95% CI 0.01 – 1.1) for clean cord tie use [55]. The three other studies reported no difference after adjusting for potential confounders, but do not present adjusted odds ratios [52,64,66].

An urban community case-control study compared use of a cord clamp versus ‘thread’ to tie the cord, and found no evidence of a difference in tetanus incidence between the two groups in multivariate analysis, where home birth and cord applications were the most important risk factors [51]. A cohort study in Tanzania did not observe any association between use of the clean birth kit thread and incidence of cord infection [56], but use was near-universal.

### 2. Hygienic cord and skin care

#### a. Combined chlorhexidine cleansing of the birth canal prior to birth and/or full body newborn cleansing immediately after birth

Vaginal washing with chlorhexidine has been shown to reduce colonization rates with Group B streptococcus in high income countries [49]. The most recent Cochrane review found no strong evidence of an effect on neonatal infections [50]. Five low and middle income country-based studies were examined which all used both vaginal and neonatal wipes. Two before-and-after hospital-based studies in Egypt and Malawi reported reductions in infection-related neonatal mortality with chlorhexidine compared to routine care [RR=0.26 (95% CI0.1 – 0.7) [67] and RR=0.33 (95% CI 0.15 – 0.70)[68]. A community-based pilot randomized controlled trial (RCT) from Pakistan of chlorhexidine versus saline vaginal and neonatal wipes did not report infection-specific outcomes and was too small to conclude anything with respect to neonatal mortality [RR=0.20 (95% CI 0.01 – 4.03)] [69]. A large randomised controlled trial (RCT) from South Africa based in a hospital with low infection rates did not find evidence of a difference in rates of sepsis with chlorhexidine vaginal and neonatal wipes compared to external genitalia water wipes and neonatal chlorhexidine foot wipes RR=0.95 (95% CI 0.76 – 1.19) [70]. Consistent with this finding, a recent large RCT based in three hospitals in Pakistan, which compared chlorhexidine vaginal and neonatal wipes to saline placebo wipes, found no difference in the primary study outcome (7 day neonatal mortality or neonatal sepsis) between the groups RR=0.91 (95%CI 0.67 – 1.24) or overall neonatal mortality at 28 days RR=0.98 (95% CI 0.68 – 1.41) [71].

A large community-based cluster RCT of skin cleansing with chlorhexidine (0.25%) as soon as possible after birth without vaginal cleansing did not find evidence of a reduction in neonatal mortality among all treated infants (RR=1.04; 95% CI:0.87 – 1.24). In a sub-analysis among low birth weight infants, those wiped with chlorhexidine experienced lower neonatal mortality than the placebo group (RR: 0.72; 95% CI: 0.55–0.95) [72].

#### b. Chlorhexidine applied to the cord postnatally

In a RCT, chlorhexidine cleansing of the cord in rural Nepal reduced all-cause neonatal mortality (RR = 0.66 [0.46 – 0.95]) and sepsis-specific neonatal mortality (RR=0.69 (95% CI 0.4 – 1.18) if applied within the first 24 hours [73]. Chlorhexidine cleansing also reduced mild (RR=0.68 (95% CI 0.58 – 0.80), moderate (RR=0.46 (95% CI 0.36 – 0.59) and severe cord infection (RR=0.25 (95% CI 0.12 – 0.53), signs that are related to subsequent risk of mortality [12]. A follow-up efficacy trial in northeast Bangladesh and an effectiveness trial in rural Pakistan have been completed and will be reported shortly. The effectiveness of this intervention is currently being evaluated in two randomized trials in sub-Saharan Africa.

#### c. Other postnatal cord antimicrobial applications

**Figure 5:**
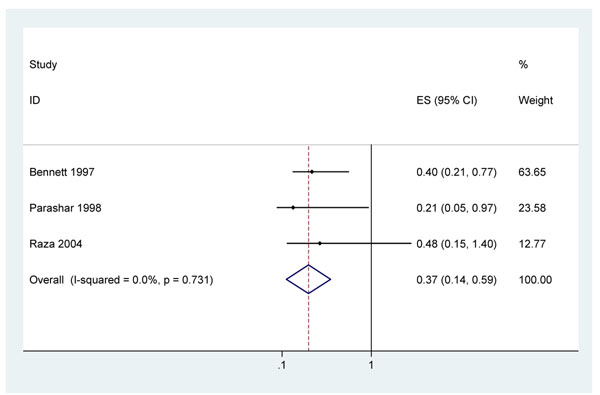
Meta-analysis of the effect of antimicrobial applications (antibiotics or disinfectants) to cord on neonatal tetanus

Four case-control studies reported that cord applications of topical antimicrobials were associated with reduced neonatal tetanus incidence compared with dry cord care. The reported associations found for topical antibiotics were [aOR=0.21(95% CI 0.05-0.97))][62] and [aOR=0.37 (95% CI 0.25 – 0.74)][74]); for disinfectants [aOR=0.69 (95% CI 0.41 – 1.19)] [62]; and for any antimicrobial [aOR=0.4 (95% CI 0.21 – 0.77)][75] and [aOR=0.48 (95% 0.15 – 1.4)] [51], based on author’s definitions. Combining these studies results in a pooled estimate aOR for the effect on neonatal tetanus of any antimicrobial to the cord of 0.37 (95% CI 0.14 – 0.59) (Figure 5). Regular antimicrobial applications during the postnatal period were associated with reduction in tetanus [aOR=0.38 (p=0.026)] [75].

#### d. Avoidance of harmful postnatal cord applications

Evidence from case-control studies supports an association between neonatal tetanus and applications of cow dung or ash to the cord [aOR=2.31 (95% CI 1.03 – 5.03)][62], traditional applications (mustard oil, ghee or surma) to the cord [aOR=5.1 (95% CI 2.7 – 9.7)] [51], shea butter [aOR 6.4 (95% CI 1.6 – 21.4)] [76] and ghee (in particular cow’s milk ghee) [aOR=1.94 (95% CI 1.07 – 3.53)] [74].

Consistent with the effect on neonatal tetanus, in one cohort study in Nepal, mustard oil application was associated with an increased risk of cord infection [aRR=1.29 (95% CI 1.11 – 1.51)] [11]. However, in a study in The Gambia application of shea butter was associated with reduced all-cause neonatal mortality [aRR=0.32 (95% CI 0.13 – 0.76)] [5].

#### e. Other postnatal skin applications including emollients

Two RCTs of hospitalized preterm infants of less than 33 weeks gestational age demonstrated reductions in nosocomial bloodstream infections with topical application of emollients(sunflower oil) [aRR=0.46 (95% CI 0.26–0.81) and aRR=0·59 (95%CI 0·37–0·96)) [77,78]. These studies also reported reductions in all cause neonatal mortality (sunflower oil [aRR 0.74(95% CI: 0.55–0.99) and using Aquaphor [aRR 0.67 (95% CI: 0.51–0.92)] [79]) in these preterm infants.

However, evidence of benefit is currently limited to inpatient preterm infants. There are currently no data on the benefit of emollients in term infants; nor is it known if the hospital-based findings among very preterm infants apply to those born at home. Two large community RCTs are currently being undertaken in India and Nepal, the results of which may provide further evidence on which to base a community-level recommendation.

### 3. Evidence for the effect of postnatal maternal handwashing

One cohort study from Nepal found that reported that regular maternal hand-washing in the first 14 days of life was associated with reductions in both omphalitis [aRR=0.76 (95% CI 0.60 – 0.95)] [11] and all-cause neonatal mortality [aRR= 0.56 (95% CI 0.38 – 0.82)] [21].

### Evaluations including clean birth kit use

Three studies using clean birth kits have reported effects on overall neonatal mortality. One before-and-after study reported a 22% reduction in all-cause neonatal mortality, OR 0.78 (95% CI 0.50 – 1.21), after introduction of clean birth kit use into an existing lady health worker-delivered package of antenatal/ birth care [80]. An 80% reduction in neonatal tetanus deaths [OR=0.20 (95% CI 0.01 – 4.18)] was also observed, explaining much of the reduction in neonatal mortality. However, increased tetanus toxoid immunisation coverage, from 70 to 93% during the study period, will have contributed to this reduction, and the relative contribution of the clean birth kits cannot be assessed. A quasi experimental study of a clean birth kit and education package introduced to a Masai population with constant levels of tetanus immunisation coverage indicated that mortality in the first six weeks of life was reduced in the intervention areas [unadjusted RR=0.17 (95% CI 0.13 – 0.23)]. A dramatic reduction in neonatal tetanus deaths [unadjusted RR=0.01 (95% CI 0.001 – 0.09)] was also seen [18]. In this setting, however, local practice included packing the umbilical stump with cow dung, and the baseline neonatal tetanus incidence was very high, raising concerns about the generalisability of the findings. One cluster RCT of a TBA-delivered package of antenatal, intrapartum and postnatal care with education and improved referral included clean birth kits and reported a 29% reduction in neonatal mortality (aOR=0.71; 95% CI 0.62 – 0.83) [81]. All three studies had multiple co-interventions and were hence excluded.

One further study, also reporting on neonatal sepsis and cord infection, was excluded as ‘newborn care kits’ were given, which included soap and spirits, but no blade or cord tie [4,82]. Three additional case-control studies were excluded, as they did not report adjusted OR for the effect of clean birth kits on neonatal tetanus [51,62,83].

One before-and-after study reported an 89% reduction in neonatal sepsis after the introduction of clean birth kits for home births [unadjusted OR=0.11 (95% CI 0.01 – 0.84) [84].

Four cross-sectional studies examined the association between clean birth kits and incidence of cord infection. They used different definitions for cord infection and showed marked heterogeneity. All studies compared clean birth kits users (adopters) with non-users (non-adopters). In three studies the kits were distributed free of charge by health workers [11,57,59]. In the fourth study, clean birth kits were marketed using mass media and made available for purchase at a subsidized price [58]. One study from Tanzania which reported a very low incidence of cord infection (1.7%), possibly due to a narrow case definition and the fact that suspected cases were required to attend the nearest health facility for confirmation of the diagnosis, observed a reduced incidence of cord infection in clean birth kit users [OR=0.08 (95% CI 0.03 – 0.19)] [59]. A study from Nepal reported 16% incidence of cord infection and found no evidence of a difference in cord infection between kit users and women who used a new or boiled blade to cut the cord [RR=1.09 (95% CI 0.75 – 1.43)], but benefit of kit use when compared to women who used a boiled or other blade to cut the cord on a dirty surface [aRR=0.45 (95% CI 0.25 – 0.81)] [58]. A study from Egypt reported a 8.2% incidence of cord infection, with a lower risk amongst kit users [aOR=0.42 (95% CI 0.18 – 0.97)] [57]. A study from Nepal reported that only the use of the soap was associated with a reduction in omphalitis (aRR=0.49 (95% CI 0.43 – 0.56)) [11], with no effect seen with any of the other kit components. Near-universal use of the other kit components prevented this study from assessing the associations of these components with risk of infection.

A study from Pemba, Tanzania found an increased risk of infection with use of a plastic cord cutting coin surface [56]. This study achieved near universal use of the other kit components and hence was not able to assess the associations of these components with the risk of infection.

### Overall level of evidence

#### Place of birth

Four studies report a protective association of neonatal tetanus with facility birth. No strong evidence of a relationship between place of birth and cord infections was found.

#### Hand-washing with soap prior to birth

Published data from four low or very low quality studies [60-63] are consistent in suggesting a reduction in tetanus mortality of 49% (95% CI 35 – 62%). A single study suggested benefit on all-cause neonatal mortality of 19% (95% CI 1 – 34%) [21]. Two low quality studies are consistent in suggesting a reduction in cord infection of 30% (95% CI. 20 – 39%) [11,58] (Table 3).

**Table 3:** 

		Quality Assessment						Summary of Findings				
					**Generalisability**			**Intervention**		**Control**		

**No of studies (ref)**	**Intervention**	**Design**	**Limitations of studies**	**Consistency**	**To population of interest**	**To intervention of interest**	**GRADE of evidence**	**No. events**	**No. births**	**No. events**	**No. births**	**Effect size** **(95% CI)**

***Neonatal Mortality (Tetanus Deaths):***												

4 (60 - 64),	Birth Attendant hand washing prior to delivery	1 Cohort, 4 Case Control	Very low quality	Consistent	All rural populations	Yes	Low					aOR = 0.51 (0.38 - 0.65)*

2 (55,66)	Clean delivery surface	2 Case control	Very low quality	Consistent	Uganda and Pakistan	Yes	Low					aOR = 0.07 (0.00 - 0.23)*

2 (51, 62)	Clean perineum	2 Case Control	Very low quality	Consistent	Both from Asia	Yes	Low					No association reported^

6 (51, 54, 60 - 61, 66)	Clean cord cutting tool	1 Cohort, 4 Case Control	Low quality, Co-interventions (adjusted for TT)	Heterogeneous	5 from Asia	Yes	Low					aOR 0.25 - 0.4^^

4 (52, 55, 64, 66)	Clean cord tie	4 Case Control	Very low quality	Heterogeneous	Yes	Yes	Low					single study aOR = 0.1 (0.01 - 1.1) 3 studies no association reported^

3 (51, 62, 75)	Antimicrobial cord applications	3 Case Control	Very low quality	Consistent	All from Asia	Yes	Low					aOR = 0.37* (0.14 - 0.59)

***Neonatal Mortality (Sepsis Deaths):***												

No studies identified												

***Neonatal Mortality (All Cause):***												

1 (21)	Birth Attendant hand washing with soap prior to delivery	Cohort	Co-interventions (adjusted for chlorhexidine)		Single study Nepal	Yes	Low	371	13,255	342	9123	aRR = 0.81 (0.66 - 0.99)

1 (21)	Postnatal maternal handwashing	Cohort	Co-interventions (adjusted for chlorhexidine)		Single study Nepal	Yes	Low	30	3403	427	19,592	aRR = 0.56 (0.38-0.82)

***Neonatal Sepsis incidence:***												

No studies identified												

***Cord infection/oomphalitis***												

2 (11,58)	Birth Attendant hand washing prior to delivery	1 Cohort, 1 Observ-ational		Consistent	Nepal only	Yes	Moderate	470**	9645**	421**	5990**	aRR = 0.73* (0.64 - 0.84)

1 (59)	Clean cord cutting tool	Cohort	Low quality		Single study from Tanzania	Yes	Low	48	2891	5	111	aOR = 1.1 (0.43 - 3.05)

1 (11)	Postnatal maternal handwashing	Cohort	Low quality		Single study Nepal	Yes	Low	95	2206	539	8960	aRR = 0.76 (0.60 - 0.95)

**only available for study (11)

*from pooled analysis ^studies did not report aOR^^199 NT cases, 3 studies. 3 studies report no association but did not present aOR.

#### Other clean birth practices

All studies are consistent with a beneficial effect of clean birth practices; however the data currently available are inadequate to evaluate the size of the effect of a clean cord cutting implement, clean perineum, clean cord tie or clean birth surface on mortality from neonatal sepsis. Limited data on the effects on cord infection were found, however, these were single studies providing low quality evidence and the outcome was distal to the outcome of interest (mortality from neonatal sepsis).

Low quality evidence of no association of a clean perineum with the incidence of neonatal tetanus, and a reduced incidence with a clean birth surface was found. Much heterogeneity was found in the six very low quality studies reporting the effect of a clean cutting implement and four very low quality studies reporting the effect of a clean cord tie on neonatal tetanus incidence. The overall of the evidence was low. (Table 3)

#### Cord and skin applications

Evidence from three very low quality studies suggests a 63% (95% CI 41 – 86%) reduction in neonatal tetanus mortality with antimicrobial applications to the cord. A community-based cluster RCT and two facility-based RCT have not found evidence of a benefit in mortality reduction of chlorhexidine skin applications with or without chlorhexidine vaginal cleansing [70-72]. A single cluster RCT of chlorhexidine applications to the cord found a reduction in neonatal mortality of 33% (95% CI 5 -54%)[73], but there is currently insufficient evidence to support inclusion of this intervention in LiST. (Table 4) The results of two further trials from Asia will soon be available and further analysis including these results is planned [85]. Any recommendation to change the current WHO recommendation for dry, clean cord care in low-resource settings awaits further evidence.

**Table 4:** 

		Quality Assessment						Summary of Findings				
					**Generalisability**			**Intervention**		**Control**		

**No of studies (ref)**	**Intervention**	**Design**	**Limitations**	**Consistency**	**To population of interest**	**To intervention of interest**	**GRADE of evidence**	**No. events**	**No. births**	**No. events**	**No. births**	**Effect size** **(95% CI)**

***Neonatal Mortality (Tetanus Deaths):***												

No studies identified												

***Neonatal Mortality (Sepsis Deaths):***												

3 (67, 68, 70)	Chlorhexidine vaginal and baby wipes	1 RCT, 2 Before and after	Hospital based studies	Heterogeneity	S.Africa, Malawi, Egypt	Yes	Moderate	21	10108	55	9612	

1 (73)	Chlorhexidine to cord (day 1)	cRCT ˇ	Single study		Nepal	Yes	Moderate		3134		3179	RR = 0.69 (0.40 - 1.18)

***Neonatal Mortality (All Cause):***												

1 (73)	Chlorhexidine to cord	cRCT	Single study		Nepal	Yes	Moderate	72	4924	98	5082	RR = 0.78 (0.57 - 1.07)*

1 (73)	Chlorhexidine to cord (day 1)	cRCT ˇ	Single study		Nepal	Yes	Moderate	45	3134	69	3179	RR = 0.66 (0.46 - 0.95)

1 (72)	Chlorhexidine wipes to baby	cRCT	Single study		Nepal	Yes	Moderate	264	860	263	8880	RR = 1.04 (0.87 - 1.24)

1 (72)	Chlorhexidine wipes to baby	cRCTˇ	Single study		Nepal	Low birth weight babies only	Moderate	83	2448	117	2491	RR = 0.72 (0.55 - 0.95)

1 (71)	Chlorhexidine vaginal and baby wipes	1 RCT	Single study		Pakistan	Yes	Moderate	55	2505	56	2503	RR = 0.98 (0.68 - 1.41)

***Neonatal Sepsis incidence:***												

2 (70,71)	Chlorhexidine vaginal and baby wipes	2 RCT	Hospital based studies	Consistent	S. Africa, Pakistan	Yes	Moderate	179	6,577	188	6,560	RR = 0.95^ (0.76 - 1.14)

***Cord infection/oomphalitis***												

1 (73)	Chlorhexidine to cord	cRCT	Single study		Nepal	Yes	Moderate	438	4703	638	4859	RR = 0.68** (0.58 - 0.80)

ˇsub-group analysis *adjusted for ethnic group, literacy, topical mustard oil applications ^based on pooled analysis **oomphalitis defined as redness extending to the skin at the base of the umbilical stump

Two high quality studies found evidence that topical emollients reduce nosocomial infections and one study reported a reduction in neonatal mortality in hospitalized preterm infants [77-79].

#### Clean postnatal newborn care practices

One study provided supportive evidence of an effect of maternal hand-washing in the postnatal period on all-cause neonatal mortality of 44% (95% CI 18 – 62%) [21].

#### Clean birth kit use

One very low quality study reported a reduction in neonatal sepsis of 89% (95% CI 16 – 99%) [84]. Four studies of the effect of clean birth kits on cord infection showed heterogeneous results. (Table 5)

**Table 5:** 

		Quality Assessment					Summary of Findings				
					**Generalisability**		**Intervention**		**Control**		

**No of studies (ref)**	**Intervention**	**Design**	**Limitations**	**Consistency**	**To population of interest**	**To intervention of interest**	**No. Events**	**No. births**	**No. events**	**No. births**	**Effect size** **(95% CI)**

***Neonatal Mortality (Tetanus Deaths):***											

1 (18)	CBK and education	Before and after	Low quality		Masai population*	Yes	0	1984	415	5716	RR = 0.01 (0.001 - 0.09)

1 (80)	CBK, TT plus multiple interventions	Before and after	Multiple interventions		India. Lady health worker delivered	Multiple interventions	0	1951	2	1958	OR = 0.20 (0.01 - 4.18)

***Neonatal Mortality (Sepsis Deaths):***											

No studies identified											

***Neonatal Mortality (All Cause):***											

1 (81)	CBK plus multiple interventions	cRCT	Multiple interventions		Pakistan TBA delivered	Multiple interventions	340	10092	439	19432	aOR 0.71 (0.62 - 0.83)

1 (18)	CBK and education	Before and after	Low quality		Masai population*	Yes	99	1984	1984	5716	RR = 0.17 (0.13 - 0.23)

1 (80)	CBK, TT plus multiple interventions	Before and after	Multiple interventions		India lady health worker delivered	Multiple interventions	35	1951	45	1958	OR 0.78 (0.50 - 1.21)

***Neonatal Sepsis incidence:***											

1 (84)	CBK and demonstration	Before and after	Observational		Papua New Guinea**	Yes	1	67	8	64	OR = 0.11 (0.01 - 0.84)

***Cord infection/oomphalitis:***											

3 (56, 58, 59)	CBK use	Adopters vs non-adopters	Observational	Heterogeneous	Egypt, Tanzania, Nepal	Yes					aOR 0.08-0.45

1 (11)	Use of individual items in CBK	Adopters vs non-adopters	Observational		Nepal	Yes					soap aRR = 0.49 (0.43-0.56)^

*Specific cultural practices and defined neonatal death as death occuring in first 6 weeks of life **Specific cultural practices

^ no effect of other components on multivariable analysis TT = tetanus toxoid vaccination

Due to concurrent interventions (including education, tetanus toxoid immunization, newborn care packages) and contextual factors, the generalisability of the remaining study findings reviewed is unclear. Hence, whilst the available data supports that clean birth kit use as part of a ‘package’ has an effect on neonatal mortality from sepsis and tetanus we were unable to estimate the individual contribution of clean birth kit use on this mortality reduction.

### GRADE recommendation and results of Delphi process

Low quality evidence was found for the effect of hand washing and for antimicrobial applications to the cord on mortality from neonatal sepsis and tetanus. Very low quality or no evidence was found for the other clean practices reviewed. This is counter balanced with the ethical complexity of randomised trials of what is a standard of care throughout the world. The GRADE recommendation for clean practices is strong. Given this recommendation, and as the objective of this review is to establish mortality effect estimates for clean practices with transparent methodology, a Delphi expert consensus was undertaken. The panel invited to participate included experts in obstetrics, gynaecology and newborn health representing five WHO regions (South Asia, Africa, Western Europe, North America, Latin America Caribbean), and including multiple disciplines - programme management, research, obstetrics, and paediatrics. Thirty experts participated.

Consensus was reached in the first round for all interventions to reduce neonatal mortality. Experts judged that clean birth practices at home with no skilled attendant could avert 15% (IQR 10 – 20) of sepsis-related and 30% (IQR 20 – 30) of tetanus-related neonatal deaths. Skilled attendance at home was judged to avert 23% (IQR 19 – 30) of sepsis-related and 35% (IQR 30 – 40) of tetanus-related neonatal deaths. Skilled attendance in a facility was judged to increase this to 27% (IQR 24 – 36) of sepsis-related and 38% (IQR 34 – 40) of tetanus-related deaths. Clean newborn care practices in the postnatal period were judged to avert 40% IQR 25 – 50) of sepsis-related and 40% (IQR 30 – 50) of tetanus-related deaths. (Table 6)

**Table 6:** Results from the Delphi expert consensus process

		Median (%)	Range (%)	Inter-quartile Range (%)
Effect on sepsis specific neonatal mortality	1. Effect of **clean birth practices at home without a skilled attendant** on sepsis specific neonatal mortality	15	5 – 30	10 – 20
	2. Effect of **clean birth practices at home with a skilled attendant** on sepsis specific neonatal mortality	23	10 – 50	19 – 30
	3. Effect of **clean birth practices in a facility** on sepsis specific neonatal mortality	27	5 – 60	23.75 – 36.25
	4. Effect of **clean newborn care practices at home during the postnatal period** on sepsis specific neonatal mortality	40	10 – 60	25 – 50

Effect on neonatal mortality due to tetanus	5. Effect of **clean birth practices at home without a skilled attendant** on neonatal mortality due to tetanus	30	5 – 45	20 – 30
	6. Effect of **clean birth practices at home with a skilled attendant** on neonatal mortality due to tetanus	35	5 – 50	30 – 40
	7. Effect of **clean birth practices in a facility** on neonatal mortality due to tetanus	38	5 – 80	34 – 40
	8. Effect of **clean newborn care practices at home during the postnatal period** on neonatal mortality due to tetanus	40	5 – 70	30 - 50

## Discussion

The primary finding and main limitation of this review is the lack of high or moderate quality evidence for the effect of clean birth and postnatal newborn care practices on neonatal mortality, particularly those relevant for low and middle income countries where the impact would be the greatest. In addition there is likely to be publication bias for positive studies. Even within published studies when multivariable analysis showed no association of certain clean practices with tetanus or sepsis-related outcomes, the adjusted effect size was not reported. The overall quality of evidence for impact of clean birth and postnatal newborn care practices reviewed on cause-specific mortality is very low. However as there is strong biological plausibility and this is an accepted standard of care, and randomized controlled trials would be considered unethical, the GRADE recommendation for these practices is strong.

The size of the effect of clean birth and postnatal care practices is important to quantify, and to our knowledge this is the first such estimate. Following the rules established by CHERG for interventions with a strong GRADE recommendation[44], but low level of evidence , a Delphi expert process was undertaken. We included a range of experts with wide geographic representation (geographic region, low-middle and high income settings) and expertise (clinical, epidemiology, obstetrics, neonatology). They estimated moderate benefits of clean practices; specifically that clean birth practices may avert between 15 - 27% of neonatal sepsis deaths and 30 – 38% of neonatal tetanus deaths. Greater benefits were estimated for clean birth practices by a skilled birth attendant and in a facility compared to clean birth practices at home with no skilled attendant. Clean newborn care practices were estimated to prevent 40% of sepsis and tetanus neonatal mortality. In total combined clean facility birth and newborn care practices are estimated to avert two thirds of neonatal sepsis deaths and over three quarters of neonatal tetanus deaths when compared to home birth and postnatal care with no clean practices (Table 7).

**Table 7:** Cause-specific mortality effect and quality grade of the estimate for the effect of clean birth and newborn care practices on neonatal deaths from sepsis and tetanus for use in LiST

***Cause-specific mortality to act on:***
Neonatal deaths from sepsis and tetanus
***Cause-specific estimate of effect:***
Reduction in neonatal deaths from sepsis of 15% with clean birth practices at home with no skilled attendant, 23% with a skilled attendant at home and 27% in a facility.
Reduction in neonatal deaths from tetanus of 30% at home with no skilled attendant, 35% at home with a skilled attendant and by 38% in a facility.
Clean postnatal newborn care practices are estimated to reduce neonatal mortality from sepsis by 40% and from tetanus by 40%
***Quality of input evidence:***
Very low quality– based on Delphi panel consensus
Moderate to very low quality supporting evidence

Neonatal mortality in high income countries showed a rapid decline throughout the last century[86]. Much of this decline occurred before the introduction of immunization, antibiotics and neonatal intensive care into routine practice. In particular, improved clean practices around the time of birth, coupled with distancing the place of birth from potential soil contamination (and hence tetanus) resulted in a substantial reduction in neonatal tetanus in these countries prior to vaccine introduction [14,87]. Historical data and data from before and after studies suggest higher reductions than our panel consensus, but such studies may have been undertaken in settings with higher risk behaviours – eg the Masai who traditionally placed cow dung on the umbilical cord. Overall our estimates are likely to be conservative.

Few would disagree with the principles of clean birth and postnatal care for all babies, including those born at home. The benefits of clean care are likely to be positive with minimal plausible adverse effects of the practices per se; however several implementation questions remain important. What is the most appropriate method to promote behaviour change in this area? What practices should be focused on? And if practice is changed, what is the actual cost, the opportunity cost and the likely effect on lives saved of rolling out the promotion method as a policy? Strategies to improve uptake of clean birth and postnatal care practices include community-based behaviour change (including women’s groups), health worker/birth attendant training and specific vehicles such as clean birth kits. Possible unintended adverse effects of these strategies may exist e.g. dis-incentivising facility birth. (Table 8) There is increasing evidence from evaluations of packages which include the promotion of clean birth and postnatal care practices as package components, showing increased uptake of clean practices in the intervention groups [4,30,32,82,88-93]. However as the packages are intended to affect multiple behaviours at once, it is difficult to tease out the effect of each intervention, particularly the effects of clean birth and postnatal care practices in the early postnatal period.

**Table 8:** Addressing the knowledge gaps for clean practices at birth and in the postnatal period

***Analysis of existing datasets***
Analysis of existing data sets to examine the relationship between clean birth practices, use of clean birth kits and neonatal mortality/ morbidity, with improved controlling for confounding variables.
***Examination of implementation experiences***
Examination of implementation approaches for the promotion of behaviour change in relation to clean practices, particularly to examine whether certain strategies for clean birth kits distribution may act as an incentive or disincentive for facility birth.
***New studies***
New research studies for example well designed randomised controlled trials of implementation strategies to improve clean birth and postnatal practices assessing benefits, feasibility, costs and potential negative effects of different strategies e.g. education, media, community mobilisation, clean birth kits.

One potential vehicle for promoting clean birth and postnatal care practices is the clean birth kit. In some settings, e.g. in conflict or humanitarian emergencies, or in settings where there is currently low coverage of facility birth, a working group of over 35 experts from multiple disciplines concluded that clean birth kits are to be recommended as long as they do not act as a disincentive for facility birth [94]. The concept of a clean birth kit has been promoted for many years, and clean birth kits have been shown to be acceptable in several populations and may be important in areas where commodities are the key constraint [58,59,96]. In study settings, clean birth kits changed behaviours directly related to the physical components of the kits [58,95], but not to more distal newborn practices depicted in the accompanying education leaflet (e.g. immediate breast feeding and wrapping of the newborn) [58]. Many families and healthcare workers are not aware of the benefits of clean practices for newborns and often coverage of these practices is low [24,27,28,96-99] and influenced by local culture, especially for cord care [22-24]. Community education and birth attendant training are both associated with a change in practice to cutting the cord with a clean blade and tying the cord with a clean tie [23,99]. Few countries have national data regarding coverage of clean birth kits from DHS, and only Nepal has comparable population level trend data [100]. Despite fairly extensive social marketing, clean birth kit use remains low (18% in 2006). Social marketing of insecticide treated bed nets for malaria in very low income communities may lead to only moderate coverage benefits, compared to free provision. Some evidence suggests that the role of a clean birth kit may be less important in communities that already have relatively high use of clean blade and hand washing[58]. Clean birth kits could be adapted to include additional components, but the benefit of adding any additional items must be weighed up against the increased cost and the appropriate use and effectiveness of these items. A recent analysis suggested that locally made clean birth kits linked with programs to improve clean practices are highly cost effective with an estimated US$215 per neonatal life saved [94]. The added benefit and cost of clean birth kit promotion compared to behaviour change strategies alone requires more analysis and evaluation.

## Conclusion

While clean birth and postnatal care is widely accepted, there is understandably low-quality evidence for the effect of these interventions especially in low income settings. However, since there is strong biological plausibility and given that clean practices are an accepted standard of care, the GRADE recommendation is strong. Our Delphi expert consensus process judged that clean birth practices at home with no skilled attendant could reduce neonatal sepsis deaths by 15% and tetanus deaths by 30%. The panel judged that clean birth practices in a facility would reduce neonatal deaths from sepsis by 27% and tetanus by 38%. Postnatal newborn care practices were considered to have a higher effect on neonatal mortality with 40% reduction in both sepsis and tetanus deaths.

More research is needed particularly on the content and quality of care during the early postnatal period. Given that most evidence to date is from South Asia, the results of ongoing studies in Africa are of especial importance. Use of standard definitions and outcome case definitions would improve future attempts at evidence synthesis. In addition population-based coverage data are lacking for clean birth practices or for use of clean birth kits.

Clean practices at birth and in the postnatal period could prevent many needless deaths, especially in settings with high baseline neonatal mortality and where the majority of births still take place at home, although in many facilities in low income settings, hygienic practices may also be sub optimal. The benefits of a clean birth have been recognised for centuries and if this basic and feasible action was achieved for every mother and baby of the 135 million births each year, over 100,000 lives could be saved each year [94].

## Authors’ contributions

JL planned the review with HB who undertook the searches and abstraction. HB and JL drafted the manuscript. ACL contributed to the design of the Delphi process. HB produced the meta-analysis. SC provided statistical support. All authors contributed to the data review and to the manuscript.

## Funding

This work was supported in part by a grant to the US Fund for UNICEF from the Bill & Melinda Gates Foundation (grant 43386) to “Promote evidence-based decision making in designing maternal, neonatal and child health interventions in low- and middle-income countries”, and by a grant to Save the Children USA from the Bill & Melinda Gates Foundation (Grant 50124) for "Saving Newborn Lives".

## Competing interests

The authors all declare no conflict of interest.

## Supplementary Material

Additional File 1 Delphi form Click here for file

Additional File 2 Detailed GRADE table Click here for file
