# Supplementary material for: Clean birth and postnatal care practices to reduce neonatal deaths from sepsis and tetanus: a systematic review and Delphi estimation of mortality effect
Source: BMC Public Health. 2011 Apr 13;11(Suppl 3):S11. doi: 10.1186/1471-2458-11-S3-S11 (PMC3231884; doi:10.1186/1471-2458-11-S3-S11)
Supplement: Additional File 1 — Delphi form [file 1471-2458-11-S3-S11-S1.docx]

### **Lives Saved Tool (LiST)** Expert consensus panel via a Delphi process

**Estimates of the effectiveness of hygienic birth and newborn care practices on neonatal mortality from sepsis and tetanus and maternal mortality from sepsis**

### [Follow up to Clean Birth Kit Workshop London 25-26^th^ March 2010 co-hosted by Saving Newborn Lives/Save the Children and Immpact (University of Aberdeen)] PLEASE REPLY BY MAY 13^th^ 2010

**Background to LiST**

The Lives Saved Tool (LiST) is based on *The Lancet* Child Survival and Neonatal Survival series modelling of lives saved and is now built into a widely accepted free demographic software package (Spectrum^TM^) and includes maternal lives saved analysis. LiST incorporates recent mortality rates by country and cause of death data for mothers, newborns and children and models the impact of increasing coverage of individual interventions on the reduction of deaths due to specific causes. Stillbirths will soon be included in the model. The user is given a menu of interventions preset with estimates of current national coverage levels (eg from Demographic and Health Surveys), and then the user sets coverage targets for each intervention by year up to 2015. The increases in coverage are linked to cause-specific mortality effect estimates for each intervention. Based on the effectiveness of the intervention and the change in coverage the number of deaths prevented is then calculated. The model assumes that each death is due to a single cause and takes account of the fact that a death can only be prevented once, and avoids double counting of lives saved. The tool and manuals can be downloaded at: [**h**ttp://www.healthpolicyinitiative.com/index.cfm?id=software&get=Spectrum](http://www.healthpolicyinitiative.com/index.cfm?id=software&get=Spectrum)

The mortality effect estimates used in the tool are based on a consistent literature review process being led by the UN’s Child health Epidemiology Reference Group (CHERG) using an adapted version of the WHO GRADE criteria to evaluate the quality of evidence, conducting meta-analyses of intervention effect size where appropriate. For widely accepted interventions with low level of evidence, expert opinion is being sought in order to arrive at effect estimates. So that the assumptions and inputs are in the public domain the detailed technological basis, cause-specific mortality reviews and effect sizes used in the LiST tool will be published in open access journals and the first batch were released in International Journal of Epidemiology (April 2010).

**Background to this Delphi process**

Given that it would be unethical to undertake randomized trials of hygienic birth and newborn care practices, high quality evidence is lacking to quantify the effect of these interventions on maternal deaths due to sepsis and neonatal deaths due to sepsis or tetanus Where evidence exists, a major challenge is that it is complex to separate the effect of the hygienic practices or the use of clean birth kits from other concurrent interventions in these studies such as tetanus immunization or other health promotion.

You have been approached to participate in this Delphi process because of your expertise in the area of hygienic birth and newborn care practices. We are asking you to answer 11 questions on the following pages. We will collate the responses. If there is strong consensus on the first round of responses, a second round of questions will not be necessary but in many cases two or even three rounds of questions may be required.

**Expert opinion requested from you**

We are asking you to estimate the percentage of maternal and neonatal lives saved by 2 separate hygienic interventions ‘Clean birth practices’ and ‘hygienic newborn care’ (after birth/postnatal)(see table on page 3).

This estimate does NOT include any other interventions during labour and delivery or the postnatal period e.g. antibiotic treatment of maternal or neonatal infections, or preventive interventions eg exclusive breastfeeding, or antibiotics for prolonged rupture of membranes. Also excluded are other new interventions which currently have insufficient evidence to support inclusion in LiST tool e.g. chlorhexidine.

**Causes of death to act on**: The estimate refers to percentage reduction in cause-specific deaths for each of the interventions. In LiST, the data is preloaded for each country with the baseline number of deaths due to the following causes. Estimated mortality reduction will directly link to these causes of death defined using UN/CHERG case definitions:

1. ***Severe neonatal infections - sepsis, meningitis*** (pneumonia not included)
2. ***Neonatal tetanus.***
3. ***Maternal sepsis.***

**Mortality effect:** We request that you provide your best estimate of the % of maternal or neonatal deaths that could be prevented if the proposed intervention had been available to all who died from this cause. Note this is not the overall reduction in MMR or NMR but the reduction just for that specific cause. Eg for maternal deaths around 10% in SSA and South Asia are due to maternal sepsis and you are estimating the reduction only for deaths allocated to this specific cause so a large effect size will still have a relatively small effect on total MMR. The comparison group you should imagine is an unattended home birth without ‘clean birth’ or ‘hygienic newborn care’ practices. Your estimates for effectiveness should reflect the benefit you would expect in a “typical,” developing country setting as opposed to efficacy under optimal conditions in a research study. Constant rates of other interventions e.g. tetanus toxoid immunization should be assumed.

**Brief summary of evidence**

**MATERNAL mortality from sepsis and effect of hygienic (clean) birth practices on:**

Maternal mortality ratio due to sepsis ranges from 0.2 in developed countries to 80 deaths per 100,000 live births in Africa. This represents more than 99.9% reduction which is due to both primary prevention and treatment. Historical data from Austria suggests that hand washing with chlorine in hospitals statistically significantly reduced maternal mortality by around 80% (RR=~0.20).

Evidence re prevention *ranges between 62 and 83% reduction* of incidence of infection:

1. **Bathing** by women prior to delivery reduced clinical diagnosis of sepsis based on fever, lower abdominal pain, and/or foul lochia by 69% (Tanzania, step wedge observational study AOR 0.31 (0.18-0.54)).
2. Use of a **clean birth kit** at home reduced clinical diagnosis of sepsis based on fever, lower abdominal pain, and/or foul lochia by 62% (Tanzania, step wedge observational study (AOR=0.38 (0.21-0.71))
3. **Hand washing** by traditional birth attendants reduced clinical diagnosis of sepsis based on fever, lower abdominal pain, and/or foul lochia by 80% (Tanzania, step wedge observational study (OR=0.2 (0.05-0.84))
4. **Three-day TBA training, clean birth kit distribution, lady health worker support, and obstetric outreach care** reduced fever or foul-smelling lochia or both by 83% (Pakistan RCT (OR= 0.17 (95% CI 0.13-0.23)

**NEONATAL mortality and effect of hygienic birth and newborn care practices**

Summary of systematic review findings for specific preventative practices:

1. **Preventive practices associated with a reduced risk of neonatal tetanus (**range 49% to 93%):
   1. **Hand washing** by birth attendant with soap prior to delivery in observational studies was associated with a 49% reduction in neonatal tetanus (1 cohort, 4 case-control studies – pooled, adjusted OR=0.51(95%c.i.0.38-0.65)),
   2. **Clean delivery surface** (very limited evidence. 2 case-control studies – very low quality adjusted OR=0.07 (95%c.i 0.00 – 0.23), but significant concerns about the robustness and generalisability of these findings)
   3. **Clean cord cutting implement** (1 cohort, 2 case-control studies adjusted OR=0.34 (95%c.i. 0.19 – 0.48))
   4. **Antimicrobial cord applications** (3 case-control studies adjusted OR=0.37 (0.14 - 0.59))
2. **Practices associated with a reduced risk of neonatal sepsis mortality/morbidity –**
   1. **Hand washing** by birth attendant with soap prior to delivery was associated with a 19% reduction in all-cause neonatal mortality (1 cohort study – adjusted RR=0.81 (95%c.i.0.66 - 0.99)) and a 30% reduction in omphalitis (1 cohort, 1 cross-sectional study - pooled estimate RR=0.70 (95%c.i.0.61 - 0.80)).
   2. **Three-day TBA training, clean birth kit distribution, lady health worker support, and obstetric outreach** reduced NMR by ~20% but given multiple interventions, the role of clean practices cannot be quantified.

All studies were of low quality or involved heterogeneous intervention packages including clean birth kits. Thus, we are requesting your expert opinion on the extent to which hygienic birth and newborn care practices can reduce mortality, compared what would be expected in the absence of these practices.

Please enter your answers in space provided, add your name (leave blank if you do not wish to be named)

and e-mail your responses back to Hannah Blencowe [hblencowe@gmail.com](mailto:hblencowe@gmail.com) *PLEASE REPLY BY MAY 13^th^ 2010*

Your name: _____________ Email: _________________________________

Affiliation: _________________________ [Leave all blank if you prefer to submit anonymously]

**THANK YOU FOR YOUR CONTRIBUTION TO THIS DELPHI PROCESS!**

**SPECIFIC QUESTIONS TO ANSWER - PLEASE FILL YOUR ANSWERS ON THE MARKED SPACE IN THE BOX**

The comparison group is an unattended home birth without ‘clean birth’ or ‘hygienic newborn care’ practices

|  | **Intervention** | **Estimated cause-specific mortality reduction (%)** |
| --- | --- | --- |
| **HOME BIRTH – NO SKILLED ATTENDANT** | 1) If **100 babies** are currently dying from **severe infection** (sepsis, meningitis), how many would be saved with clean birth practices at home with no skilled attendant? Bearing in mind that around 30% of such deaths are early onset sepsis (i.e. first 3 days) and may be related to labour/birth although there are other causes of early onset sepsis e.g Group B strep. | ___ % |
|  | 2) If **100 babies** are currently dying from **neonatal tetanus** how many would be saved with clean birth practices at home with no skilled attendant? Bearing in mind that around 40% of such deaths may be directly related to intrapartum care practices and the remainder are acquired after birth during the postnatal period. | ___ % |
|  | 3) If **100 mothers** are currently dying from **sepsis,** how many would be saved with clean birth practices at home with no skilled attendant during labour? | ___ % |
| **HOME BIRTH –SKILLED ATTENDANT** | 4) If **100 babies** are currently dying from **severe infection** (sepsis, meningitis), how many would be saved with clean birth practices at home with a skilled attendant? Bearing in mind that around 30% of such deaths are early onset sepsis (ie first 3 days) and may be related to labour /birth although there are other causes of early onset sepsis eg Group B strep. | ___ % |
|  | 5) If **100 babies** are currently dying from **neonatal tetanus** how many would be saved with clean birth practices at home with a skilled attendant? Bearing in mind that around 40% of such deaths may be related to intrapartum care practices and the remainder are acquired after birth during the postnatal period. | ___ % |
|  | 6) If **100 mothers** are currently dying from **sepsis,** how many would be saved with clean birth practices at home with a skilled attendant during labour? | ___ % |
| **FACILITY BIRTH** | 7) If **100 babies** are currently dying from **severe infection** (sepsis, meningitis), how many would be saved with clean birth practices in a facility during labour? Bearing in mind that around 30% of such deaths are early onset sepsis (ie first 3 days) and may be related to labour and birth although there are other causes of early onset sepsis eg Group B strep and vaginal carriage. | ___ % |
|  | 8) If **100 babies** are currently dying from **neonatal tetanus** how many would be saved with clean birth practices in a facility during labour? Bearing in mind that around 40% of such deaths may be related to intrapartum care practices. | ___ % |
|  | 9) If **100 mothers** are currently dying from **sepsis,** how many would be saved with clean **birth practices in a facility** during labour? | ___ % |
| **POSTNATAL** | 10) If **100 babies** are currently dying from **severe infection** (sepsis, meningitis), how many would be saved with hygienic newborn care practices during the postnatal period? Bearing in mind that around 70% of such deaths are late onset sepsis, especially cord care practices | ___ % |
|  | 11) If **100 babies** are currently dying from **neonatal tetanus** how many would be saved with hygienic newborn care practices during the postnatal period? Bearing in mind that around 60% of such deaths are likely to be related to postnatal practices especially cord care. | __ % |
